# LncRNA Hoxaas3 promotes lung fibroblast activation and fibrosis by targeting miR-450b-5p to regulate Runx1

**DOI:** 10.1038/s41419-020-02889-w

**Published:** 2020-08-26

**Authors:** Shuang Lin, Rui Zhang, Lei Xu, Rui Ma, Liming Xu, Linghua Zhu, Jian Hu, Xiaoxia An

**Affiliations:** 1 Department of Thoracic Surgery, the First Affiliated Hospital, College of Medicine, Zhejiang University, Hangzhou, Zhejiang China; 2 Department of Internal Medicine, Hangzhou Wuyunshan Sanatorium, the Affiliated Hangzhou First People’s Hospital, College of Medicine, Zhejiang University, Hangzhou, Zhejiang China; 3 Department of Thoracic Surgery, Henan Province People’s Hospital, Zhengzhou, Henan China; 4 Department of Surgery, Zhejiang University Hospital, Zhejiang University, Hangzhou, Zhejiang China; 5 Department of Pathology, the First Affiliated Hospital, College of Medicine, Zhejiang University, Hangzhou, Zhejiang China; 6 Department of General Surgery, Sir Run Run Shaw Hospital, College of Medicine, Zhejiang University, Hangzhou, Zhejiang China; 7 Department of Anesthesiology, the First Affiliated Hospital, College of Medicine, Zhejiang University, Hangzhou, Zhejiang China

**Keywords:** Epithelial-mesenchymal transition, Long non-coding RNAs

## Abstract

Long noncoding RNAs (lncRNAs) participate in organ fibrosis and various pulmonary diseases, but its role in idiopathic pulmonary fibrosis (IPF) is not fully understood. In this study, we found lncRNA Hoxaas3 (Hoxaas3) was up-regulated in the mice model of BLM-induced PF and TGF-β1-induced fibrogenesis in lung fibroblasts (LF). Overexpression of Hoxaas3 promoted fibrogenesis, whereas Hoxaas3 inhibition attenuated lung fibrosis both in vitro and in vivo, through regulation of miR-450b-5p. Furthermore, miR-450b-5p inhibition stimulated fibrogenesis by regulating runt-related transcription factor 1 (Runx1), whereas up-regulation of miR-450b-5p alleviated fibrogenesis in LF. Mechanistically, our study showed that Hoxaas3 regulated lung fibroblast activation and fibrogenesis by acting as a competing endogenous RNA for miR-450b-5p: Hoxaas3 decreased the expression of miR-450b-5p to stimulate level and activity of Runx1 and induced fibrotic LF, whereas Runx1 inhibition alleviated the pro-fibrotic effect of Hoxaas3. In addition, Hoxaas3 was regulated by TGF-β1/Smad4 pathway as its transcriptional target. In conclusion, our study showed the role and mechanism of the TGF-β1/Smad4- Hoxaas3–miR-450b-5p–Runx1 axis for a better understanding of PF, demonstrated Hoxaas3 maybe a new diagnostic biomarker or potential therapeutic target for IPF.

## Introduction

Idiopathic pulmonary fibrosis (IPF) is a chronic, progressive, fibrotic interstitial pulmonary disease of unknown reasons, often characterized primarily by excessive deposition of extracellular matrix (ECM) proteins by activated lung fibroblasts (LF) and myofibroblasts, leading to decreased exchange and impaired pulmonary function^1,2^. It is one of the most common idiopathic interstitial pneumonia and the most commonly interstitial pulmonary disease, with an estimated incidence of 50/100,000^3–5^. The majority of IPF occurs primarily in older adults, with median survival after diagnosis limited to 3–5 years^6–8^. There is no widely accepted treatment for IPF, although the new anti-fibrotic drugs, pirfenidone, and nintedanib, can alleviate IPF progress^9^. Therefore, it is essential to explore the molecular mechanisms of IPF and to discover more effective therapeutic strategies and drugs for the treatment of IPF.

Long noncoding RNAs (lncRNAs) are transcripts of more than 200 nucleotides. They have a variety of biological regulatory functions, including proliferation, differentiation, apoptosis, immune response, etc^10–14^, and have recently attracted widespread attention. Although many lncRNAs have crucial functions in various diseases, only a few lncRNAs have been reported to participate in pulmonary fibrosis (PF). The function and mechanisms of lncRNAs in IPF regulation remain largely unknown, and further investigation into the molecular mechanism of lncRNAs for IPF may promote the development of improved therapies.

Our study aimed to explore the role and potential mechanisms of a lncRNA, Mus musculus Hoxa cluster antisense RNA 3 (lncRNA Hoxaas3, Hoxaas3) in IPF. This research is a follow-up to a previous study performed in our laboratory. Previously, we investigated its homologous gene, HOXA-AS3, which is upregulated by cisplatin therapy, and we found that HOXA-AS3 inhibition enhanced the efficacy of cisplatin. HOXA-AS3 regulated cisplatin resistance by interacting with HOXA3, including the mRNA and protein forms, then reducing its expression. Moreover, HOXA3 inhibition raised cisplatin resistance and promoted changes in epithelial–mesenchymal transition (EMT)^15^. In the current study, we found that Hoxaas3 regulated fibrogenesis by acting as a ceRNA for miR-450b-5p to regulate Runx1 in lung fibroblasts, further verifying that Hoxaas3 was a transcriptional target of the TGF-β1/Smad4 pathway. In addition, our study, for the first time, to our knowledge, demonstrated that silencing Hoxaas3 alleviated PF in vitro and in vivo. Collectively, this study elucidated the mechanism of Hoxaas3 and provided a new diagnostic biomarker or potential therapeutic target for IPF.

## Results

### Up-regulation of Hoxaas3 during PF in mice

Increasing literature has indicated that lncRNAs played an essential role in the process of PF. A BLM-treated PF model was established in mice to investigate the role of lncRNAs in IPF. This model was confirmed by H&E staining and Masson trichrome assay to detect collagen deposition (Fig. S1A) and increase of fibrosis area (Fig. S1B). Meanwhile, qPCR analysis displayed that the mRNA expression of collagen-1a1, collagen-3a1, and fibronectin were upregulated in the lung tissues from the BLM-induced model compared with tissues in the saline group (Fig. S1C–E). Furthermore, the fibrosis-related proteins (FRP) of fibronectin and a-SMA were also raised by western blot (Fig. S1F). These results indicated that the BLM-induced PF model in mice was successfully established. In addition, H&E staining and Masson trichrome assay for sections of the heart (Fig. S2A), liver (Fig. S2B), and kidney (Fig. S2C) showed no obvious fibrosis in the model of BLM-treated PF in mice.

**Fig. 1:**
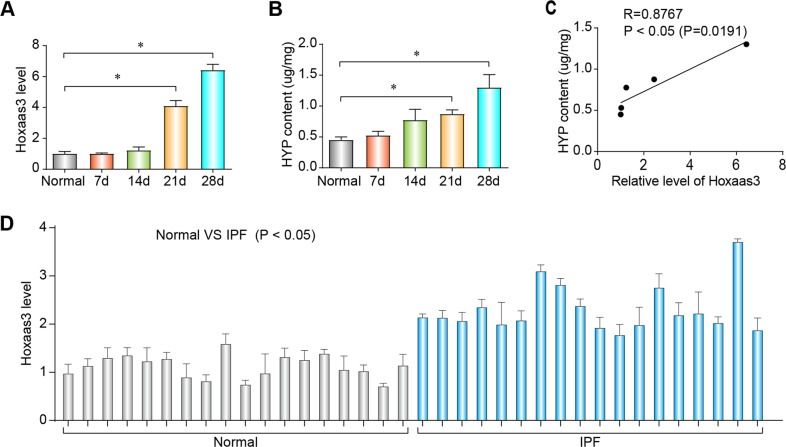
Up-regulation of Hoxaas3 during pulmonary fibrosis in mice. **a** The expression of Hoxaas3 in BLM-treated mice was detected at 0–28 days via qPCR (*n* = 6). **b** HYP content was measured at 0–28 days (*n* = 6). **c** The positive correlation between Hoxaas3 expression and HYP content in mice as determined using the Pearson correlation coefficient (*n* = 6). **d** Expression of Hoxaas3 in pulmonary fibrotic tissues of IPF patients and normal lung tissues (*n* = 18). (All data are presented as mean ± SD, *P < 0.05).

A previous study showed that lncRNAs expressed differently in lung tissues of PF model in mice and normal control mice^16^. Among these lncRNAs, we focused on Hoxaas3 for further studies. To further validate the results of microarray analysis, we used qPCR to study the expression of Hoxaas3 in BLM-induced animal models. Increased expression of Hoxaas3 was observed at 21 and 28 days compared with that in the saline group (Fig. 1a). HYP is an indicator of PF, and its expression was also up-regulated at 14 and 28 days (Fig. 1b). The correlation between Hoxaas3 and HYP was determined by the Pearson correlation coefficient. Statistical analysis showed that Hoxaas3 was positively correlated with HYP, indicating that Hoxaas3 was positively correlated with the level of PF (Fig. 1c). Furthermore, the level of Hoxaas3 was higher in pulmonary fibrotic tissues of 18 IPF patients than 18 normal lung tissues (Fig. 1d). These results indicated that Hoxaas3 level is up-regulated in the mice model of BLM-induced PF and IPF patients. Hoxaas3 may play an important role in PF.

### Silencing Hoxaas3 attenuated BLM-induced PF in mice

**Fig. 2:**
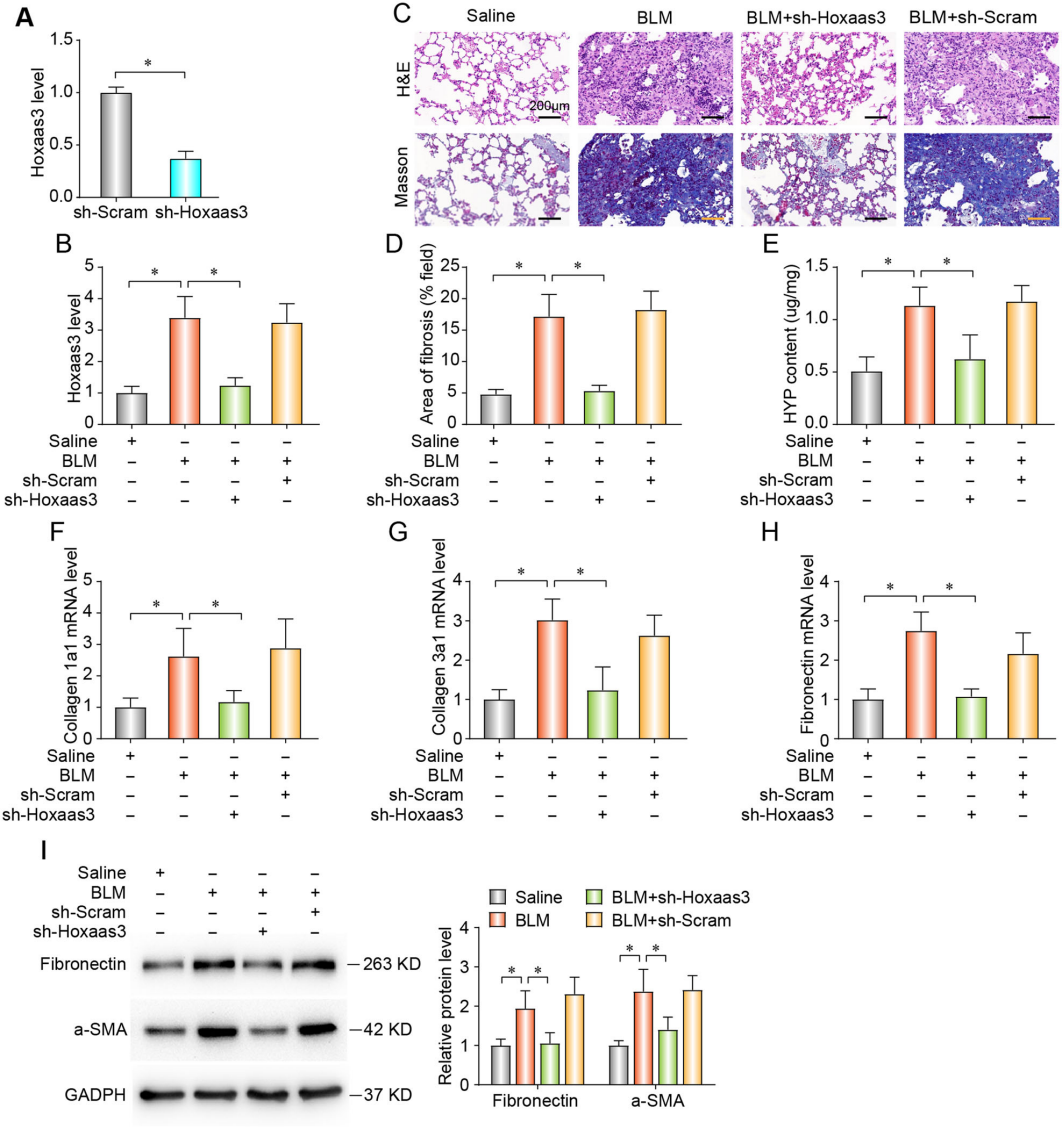
The knockdown of Hoxaas3 alleviates BLM-induced PF in mice. **a** The efficiency of sh-Hoxaas3 in fibroblasts was assessed via qPCR. **b** Intratracheal instillation of sh-Hoxaas3 decreased Hoxaas3 level compared with that in the BLM group based on qPCR. **c** Histological images and collagen deposition of the lung tissue was detected by H&E and Masson staining. **d**, **e** Fibrotic area (**d**) and HYP content (**e**) were detected in BLM-treated mice after infection with sh-Hoxaas3. **f**–**h** qPCR analysis of mRNA levels of collagen 1a1 (**f**), collagen 3a1 (**g**), and Fibronectin (**h**) in BLM-treated mice after knockdown of Hoxaas3. **i** Detection of Fibronectin and a-SMA level by western blot in BLM-treated mice after Hoxaas3 inhibition. (All data are presented as mean ± SD, *n* = 6, *P < 0.05).

To investigate the therapeutic role of Hoxaas3 in PF, sh-Hoxaas3, or sh-Scram was intratracheally injected into mice on 3 days after BLM treatment. The efficiency of the sh-Hoxaas3 knockdown was evaluated by qPCR in fibroblasts (Fig. 2a), and Hoxaas3 knockdown inhibited the up-regulation of Hoxaas3 in BLM-induced mice (Fig. 2b). Moreover, Hoxaas3 inhibition reduced collagen deposition (Fig. 2c), fibrotic area (Fig. 2d), and HYP (Fig. 2e) in the BLM-induced mice. Meanwhile, Hoxaas3 knockdown alleviated the BLM-induced up-regulation of mRNA expressions of collagen-1a1, collagen-3a1, and fibronectin (Fig. 2f–h). Furthermore, silencing Hoxaas3 eliminated BLM-treated increase of FRP, including fibronectin and a-SMA by western blot (Fig. 2i). These data suggested that Hoxaas3 inhibition attenuated BLM-induced PF in mice and that Hoxaas3 silencing is a potential treatment for PF.

### Hoxaas3 is a transcriptional target of the TGF-β1/Smad4 pathway

**Fig. 3:**
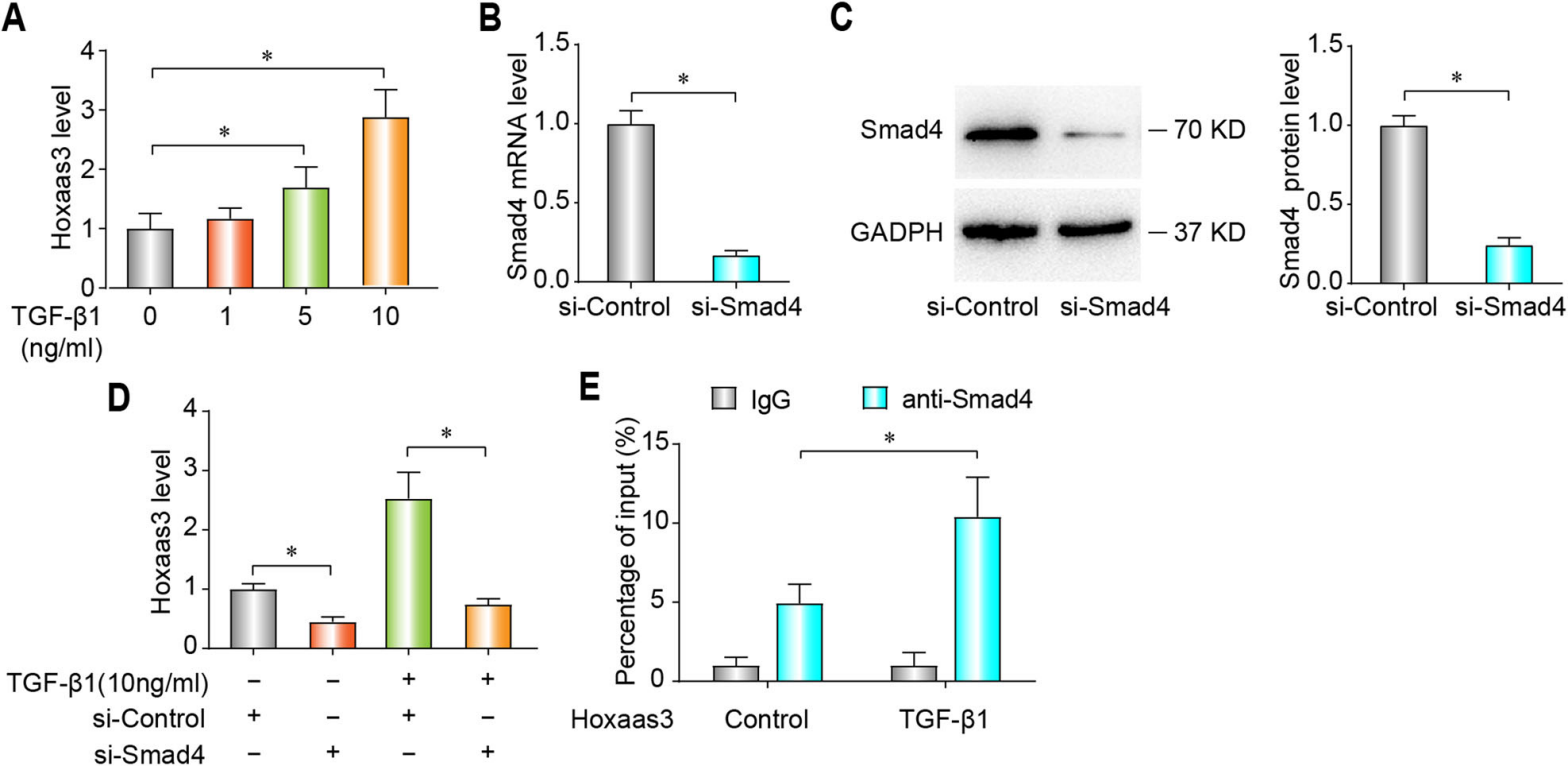
Hoxaas3 is a transcriptional target of the TGF-β1/Smad4 pathway. **a** qPCR analysis of Hoxaas3 levels in lung fibroblasts treated with TGF-β1. **b**, **c** The efficiency of the Smad4 knockdown was assessed via qPCR (**b**) and western blot (**c**). **d** qPCR analysis of Hoxaas3 after Smad4 knockdown with or without TGF-β1. **e** ChIP assays showed the level of Smad4 binding to the promoter of Hoxaas3; IgG was used as a negative control. (All data are presented as mean ± SD, *n* = 3, *P < 0.05).

In order to further clarify the signaling pathway of Hoxaas3 regulating PF, we investigated the upstream mechanism of Hoxaas3. Many researches have indicated that the TGF-β1/Smad4 signaling pathway played an important role in PF. Hoxaas3 level was found to be upregulated in cultured mouse lung fibroblasts (CMLF) treated with TGF-β1 (10 ng/ml) for 48 h in a dose-dependent manner (Fig. 3a). The PROMO online database (http://alggen.lsi.upc.es/cgi-bin/promo_v3/promo/promoinit.cgi?dirDB=TF_8.3) and JASPAR online database (http://jaspardev.genereg.net/) were used to predict Smad4 binding sequence (AGGAAAGACAT) at the promoter of Hoxaas3 with high scores. Smad4 was effectively inhibited by siRNA at both the mRNA (Fig. 3b) and the protein level (Fig. 3c). Smad4 inhibition reduced Hoxaas3 level in both control and TGF-β1-induced CMLF (Fig. 3d), indicating Smad4 may play a specific role in the stimulation of Hoxaas3 mediated by TGF-β1. ChIP assay was used to investigate whether Smad4 could bind to the promoter sequence of Hoxaas3. The level of Smad4 binding to the promoter of Hoxaas3 increased compared with the control group, IgG was as a negative control (Fig. 3e). These results showed Hoxaas3 is regulated by the TGF-β1/Smad4 pathway as its transcriptional target.

### Hoxaas3 negatively regulates the function of miR-450b-5p to promotes fibrogenesis

**Fig. 4:**
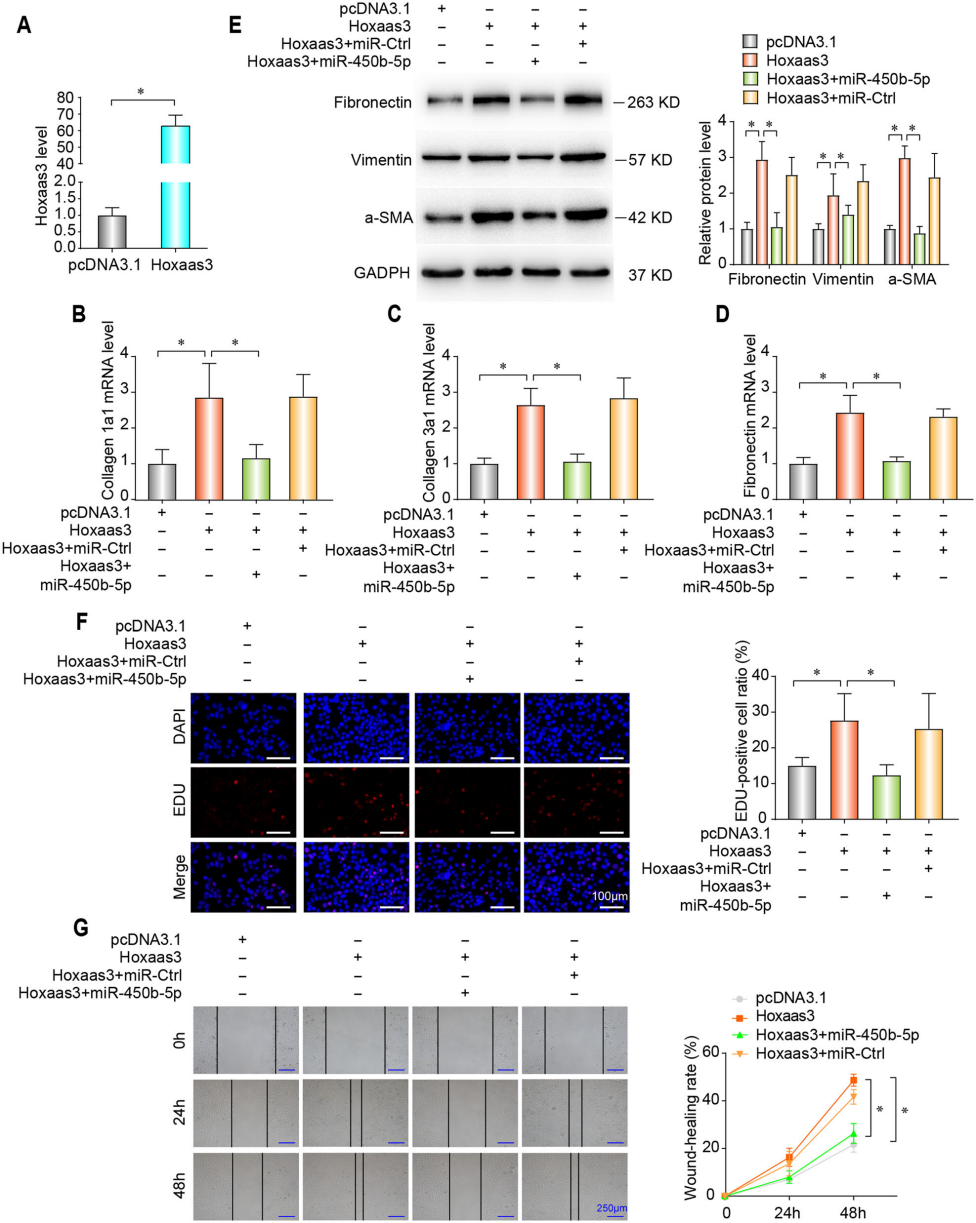
Hoxaas3 promotes fibrogenesis in cultured lung fibroblasts by inhibiting the function of miR-450b-5p. **a** The expression of Hoxaas3 was detected via qPCR after Hoxaas3 transfection. **b**–**d** qPCR analysis of mRNA levels of collagen 1a1 (**b**), collagen 3a1 (**c**), and Fibronectin (**d**) after transfection of Hoxaas3 together with miR-450b-5p. **e** Western blot analysis of expression of Fibronectin, Vimentin, and a-SMA. **f** Proliferative capacity was analyzed using an EDU assay. **g** Wound-healing assays were performed to examine cell migration. (All data are presented as mean ± SD, *n* = 3, *P < 0.05).

We next investigated the downstream of Hoxaas3 regulating PF. Bioinformatics analysis, using the databases DIANA TOOLS, was performed to search for the target miRNA that has potential binding sites for Hoxaas3. We hypothesized that Hoxaas3 contributes to PF through modulation of miR-450b-5p. To address this question, Hoxaas3 sequences were transfected into fibroblasts (Fig. 4a). Overexpression of Hoxaas3 upregulated the mRNA expression of collagen-1a1 (Fig. 4b), collagen-3a1 (Fig. 4c), and fibronectin (Fig. 4d), whereas those changes were neutralized by upregulation of miR-450b-5p. In addition, upregulation of miR-450b-5p repressed Hoxaas3-induced increase of fibronectin, vimentin, and a-SMA by western blot (Fig. 4e). Proliferation and differentiation of fibroblasts play important role in PF, promoting ECM deposition and aggravating fibrosis. Overexpression of Hoxaas3 increased cell proliferation (Fig. 4f) and migration (Fig. 4g), whereas these results were neutralized by upregulation of miR-450b-5p. All of these data indicate that Hoxaas3 promotes fibrogenesis in CMLF by repressing miR-450b-5p.

To further investigate the relationship between Hoxaas3 and miR-450b-5p, a loss-of-function method was performed. Transfection of sh-Hoxaas3 diminished TGF-β1-induced the mRNA level of collagen-1a1 (Fig. S3A), collagen-3a1 (Fig. S3B), and fibronectin (Fig. S3C), whereas that function was nearly alleviated by the miR-450b-5p inhibitor, along with changes of fibronectin, vimentin, and a-SMA by western blot (Fig. S3D). Furthermore, inhibition of Hoxaas3 decreased the TGF-β1-induced proliferation (Fig. S3E) and migration (Fig. S3F), whereas these changes were also nearly alleviated with miR-450b-5p inhibitor. These results indicated that silencing Hoxaas3 inhibited TGF-β1-induced fibrogenesis through activation of miR-450b-5p.

### Hoxaas3 regulates miR-450b-5p activity by acting as a competing endogenous RNAs (ceRNA)

**Fig. 5:**
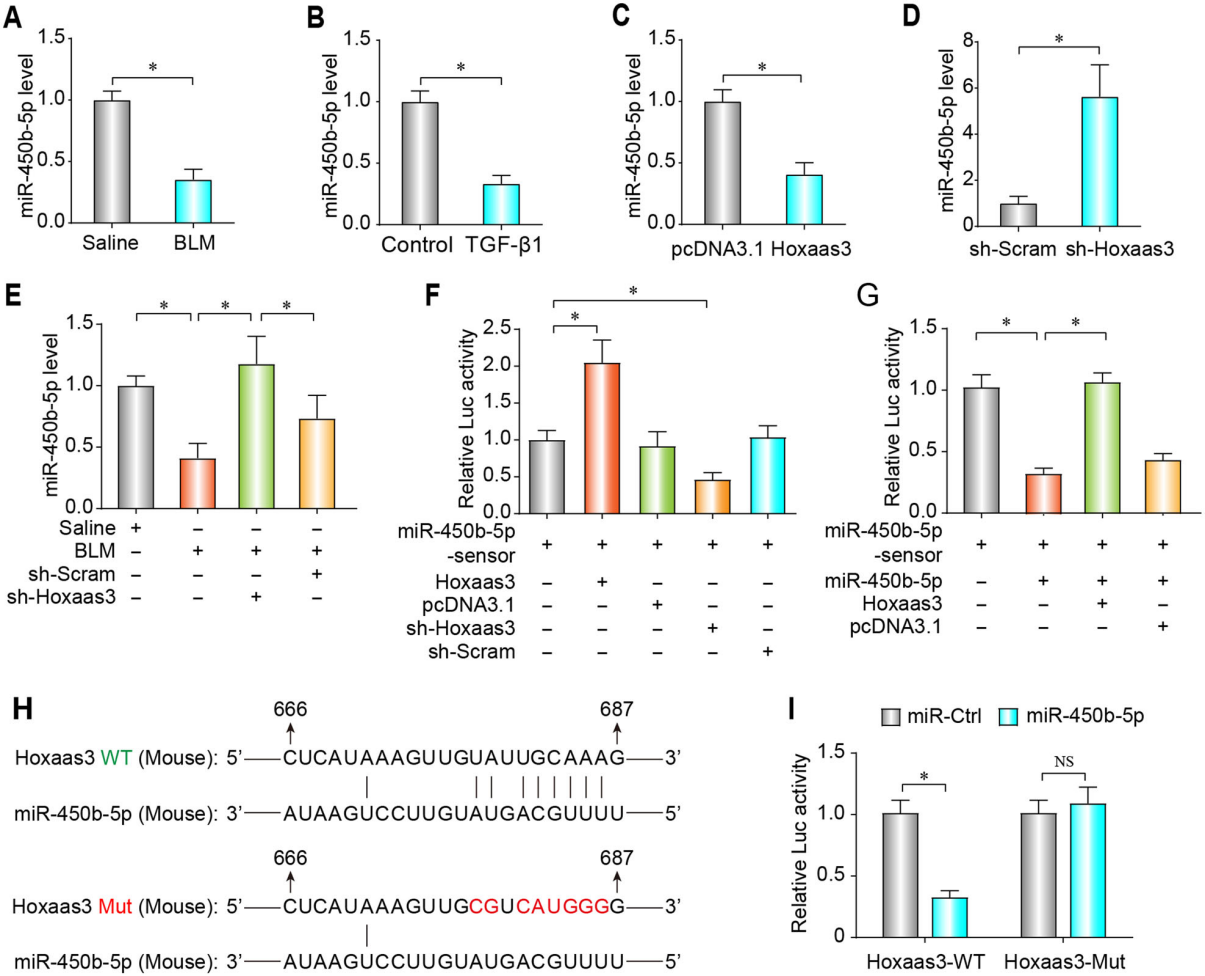
Hoxaas3 regulates expression and activity of miR-450b-5p by acting as a ceRNA. **a**, **b** qPCR analysis of miR-450b-5p expression in the lungs of BLM-treated mice (**a**) (*n* = 6) and cultured lung fibroblasts treated with TGF-β1 (**b**) (*n* = 3). **c**, **d** qPCR analysis of expression of miR-450b-5p in lung fibroblasts after Hoxaas3 overexpression (**c**) or inhibition (**d**) (*n* = 3). **e** qPCR analysis of the expression of miR-450b-5p in the lungs of mice after injection of BLM with or without Hoxaas3 knockdown (*n* = 6). **f**, **g** Hoxaas3 binds to miR-450b-5p and regulates its activity. Lung fibroblasts were co-transfected with the miR-450b-5p sensor and Hoxaas3 or sh-Hoxaas3 and its corresponding scrambled form, and luciferase activity was detected (**f**). Lung fibroblasts were co-transfected with the miR-450b-5p sensor, miR-450b-5p, and Hoxaas3 and its corresponding scrambled form, and luciferase activity was detected (**g**) (*n* = 3). **h** Predicted binding sites of Hoxaas3 and miR-450b-5p. **i** Luciferase reporter activity of vectors carrying the luciferase gene and a fragment of Hoxaas3 containing wild-type or mutated miR-450b-5p binding site (*n* = 3). (All data are presented as mean ± SD, *P < 0.05; NS, no significance).

Accumulating evidence has suggested that lncRNAs can play a role of ceRNA to bind to miRNA to regulate its activity. The expression of miR-450b-5p was found to be reduced in both the lung tissues of BLM-treated mice (Fig. 5a) and TGF-β1-treated CMLF (Fig. 5b). Forced of Hoxaas3 level decreased the miR-450b-5p level (Fig. 5c), whereas Hoxaas3 silencing increased miR-450b-5p (Fig. 5d) in CMLF. In addition, Hoxaas3 knockdown rescued the down-regulation of Hoxaas3 in BLM-treated mice (Fig. 5e).

A miR-450b-5p sensor luciferase vector, consisting of a perfect miR-450b-5p target sequence, which was integrated into the 3′ UTR of the luciferase gene, was designed to obtain better direct evidence of the interaction of Hoxaas3 with miR-450b-5p. Increased luciferase activity of miR-450b-5p sensor in CMLF transfected with Hoxaas3; Conversely, silencing of Hoxaas3 reduced luciferase activity of miR-450b-5p sensor (Fig. 5f). In addition, Hoxaas3 overexpression reduces miR-450b-5p’s inhibitory effect on its sensors (Fig. 5g). Further evidence came from our research using a luciferase vector to have a Hoxaas3 fragment containing wild type (Hoxaas3-WT) or mutation (Hoxaas3-Mut) binding site for miR-450b-5p (Fig. 5h). miR-450b-5p inhibited the luciferase activity with Hoxaas3-WT in HEK293T cells, but miR-450b-5p missed its inhibitory function with Hoxaas3-Mut (Fig. 5i). These data suggested that Hoxaas3 regulates the expression and activity of miR-450b-5p by acting as a ceRNA.

### miR-450b-5p regulates fibrogenesis by directly targeting the expression of Runx1

Next, miR-450b-5p mimic was transfected into CMLF to investigate whether miR-450b-5p has an anti-fibrotic effect during PF. Overexpression of miR-450b-5p neutralized TGF-*β*1-induced increase of the collagen-1a1 (Fig. S4A), collagen-3a1 (Fig. S4B), and fibronectin (Fig. S4C) by qPCR, along with the expression of fibronectin, vimentin and a-SMA by western blot (Fig. S4D). Furthermore, forced expression of miR-450b-5p abolished the TGF-*β*1-treated cell proliferation (Fig. S4E) and migration (Fig. S4F) of CMLF.

**Fig. 6:**
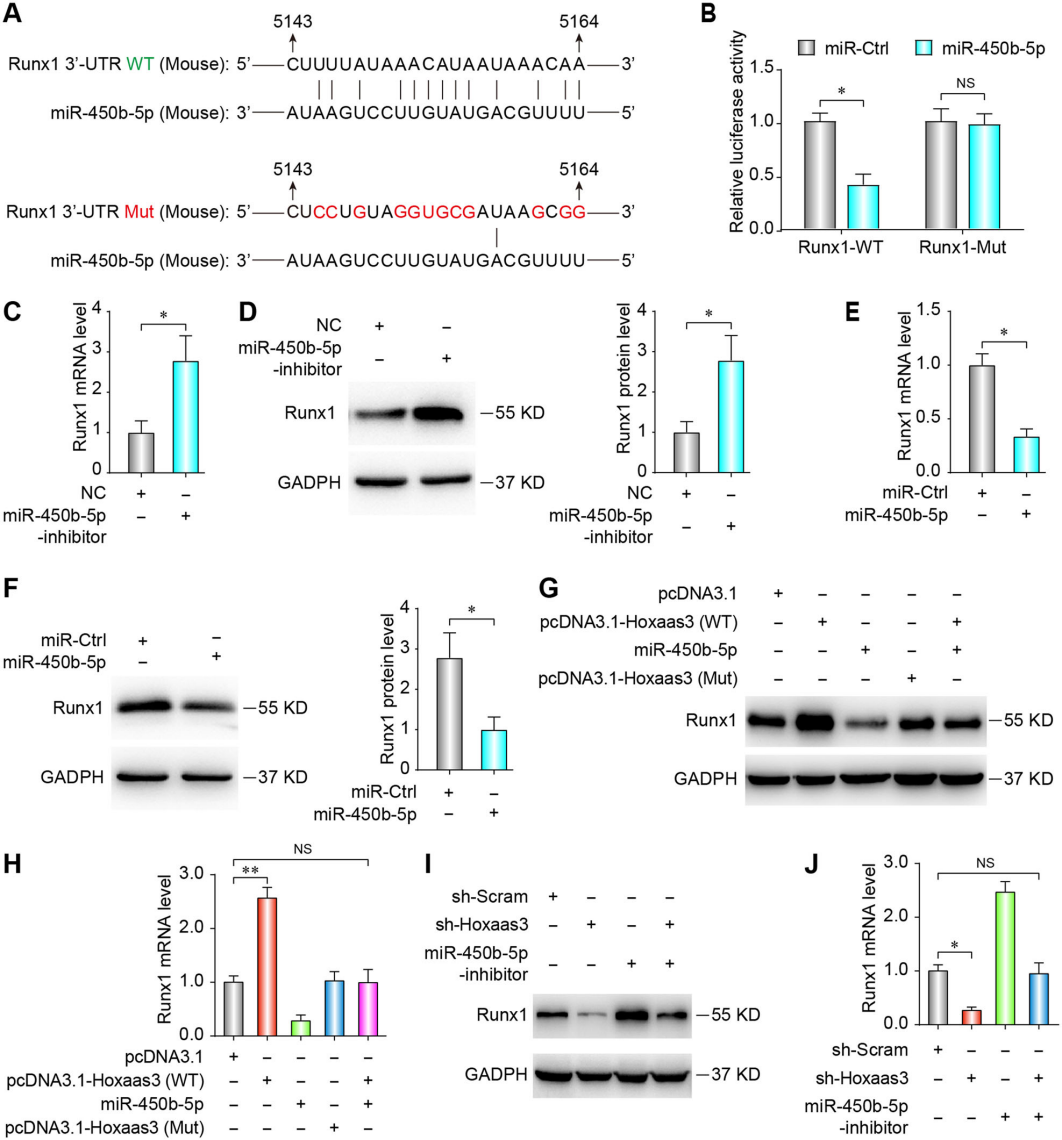
Runx1 is a direct target of miR-450b-5p. **a** Complementary sequences of miR-450b-5p and Runx1 are shown for the mouse, rat, and human genes. **b** Measurement of luciferase reporter assay demonstrates the interaction between miR-450b-5p and Runx1-WT or Runx1-Mut (fragment with the Runx1 3′-UTR containing the wild-type or mutant miR-450b-5p binding sites). **c**, **e** qPCR assays were performed to detect Runx1 mRNA expression after transfection of miR-450b-5p mimics or a miR-450b-5p inhibitor. **d**, **f** Western blot analysis of Runx1 protein levels in lung fibroblasts after transfection of miR-450b-5p mimics or a miR-450b-5p inhibitor. (All data are presented as mean ± SD, *n* = 3, *P < 0.05; NS, no significance). **g**, **h** The protein and mRNA levels of Runx1 in lung fibroblasts after transfection of pcDNA3.1-Hoxaas3 (WT), pcDNA3.1-Hoxaas3 (Mut), miR-450b-5p mimics, or co-transfection with pcDNA3.1-Hoxaas3 (WT) and miR-450b-5p mimics. **i**, **j** The protein and mRNA levels of Runx1 in lung fibroblasts after transfection of sh-Hoxaas3, miR-450b-5p inhibitor, or co-transfection with sh-Hoxaas3 and miR-450b-5p mimics.

In order to further explore the anti-fibrotic mechanism of miR-450b-5p, Targetscan, miRanda and miRWalk databases were performed to find potential targets of miR-450b-5p. To further explore the anti-fibrotic mechanism of miR-450b-5p, we used Targetscan, miRanda, and miRWalk databases to find potential targets for miR-450b-5p. In that way, Runx1 3’ UTR was found to have perfect matching sites for miR-450b-5p. A luciferase reporter vector was established, containing wild type (Runx1-WT) or mutation (Runx1-Mut) binding site of miR-450b-5p (Fig. 6a). Luciferase assay showed that miR-450b-5p reduced Runx1-WT luciferase vector activity, but there was no relationship between miR-450b-5p and Runx1-Mut vector (Fig. 6b). Furthermore, miR-450b-5p inhibition increased the Runx1 expression (Fig. 6c, d), whereas upregulation of miR-450b-5p repressed the Runx1 level both at mRNA and protein levels (Fig. 6e, f). The results showed that miR-450b-5p directly suppressed Runx1 in a transcriptional manner.

In the rescue experiment, Hoxaas3 (WT) overexpression induced upregulation of Runx1 was recovered by co-transfection with miR-450b-5p mimics at both protein and mRNA levels (Fig. 6g, h). On the other hand, sh-Hoxaas3 induced downregulation of Runx1 was recovered by co-transfection with miR-450b-5p inhibitor at both protein and mRNA levels (Fig. 6i, j). Thus, Hoxaas3 regulates Runx1 by directly adsorbing miR-450b-5p.

**Fig. 7:**
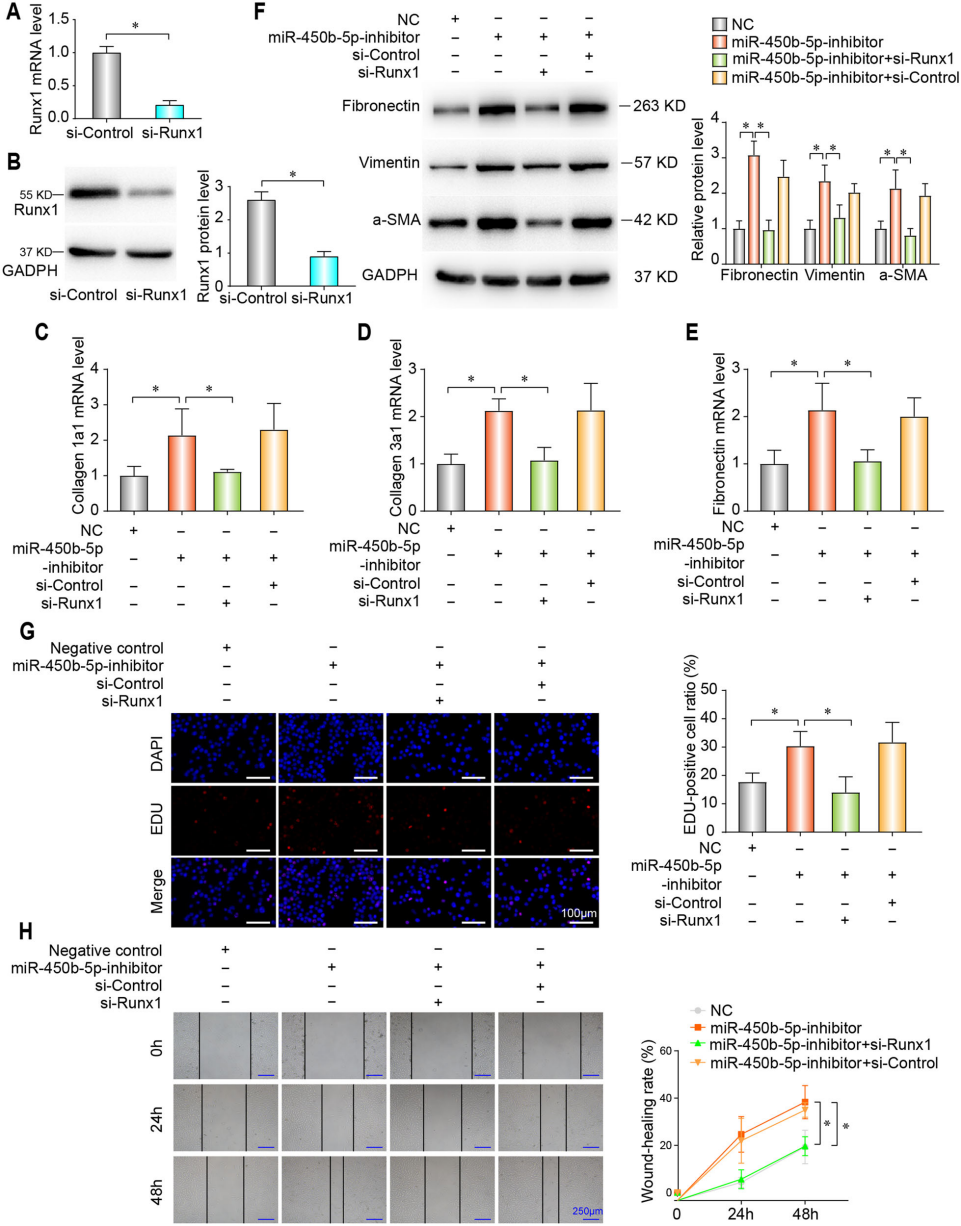
miR-450b-5p inhibition leads to fibrogenesis by regulating Runx1. **a**, **b** qPCR and western blot analysis of Runx1 mRNA (**a**) and protein (**b**) level in lung fibroblasts after transfection of miR-450b-5p inhibitor together with Runx1 inhibition. **c**–**e** qPCR analysis of mRNA levels of collagen 1a1 (**c**), collagen 3a1 (**d**), and Fibronectin (**e**). **f** Western blot analysis of expression of Fibronectin, Vimentin, and a-SMA. (**g**) Proliferative capacity was analyzed using an EdU assay. **h** Wound-healing assays were performed to examine cell migration. (All data are presented as mean ± SD, *n* = 3, *P < 0.05).

Subsequently, we investigated the role of the Runx1 on miR-450b-5p inhibition promoting fibrogenesis. Runx1 was effectively inhibited by siRNA at both mRNA (Fig. 7a) and protein level (Fig. 7b). miR-450b-5p inhibition increase the mRNA level of collagen-1a1 (Fig. 7c), collagen-3a1 (Fig. 7d), and fibronectin (Fig. 7e), which was nearly ameliorated by Runx1 knockdown, along with the expression of fibronectin, vimentin, and a-SMA by western blot (Fig. 7f). Furthermore, miR-450b-5p inhibitor increased proliferation (Fig. 7g) and migration (Fig. 7h), whereas Runx1 inhibition effectively alleviated these effects.

Hoxaas3 contributes to PF by regulating Runx1

**Fig. 8:**
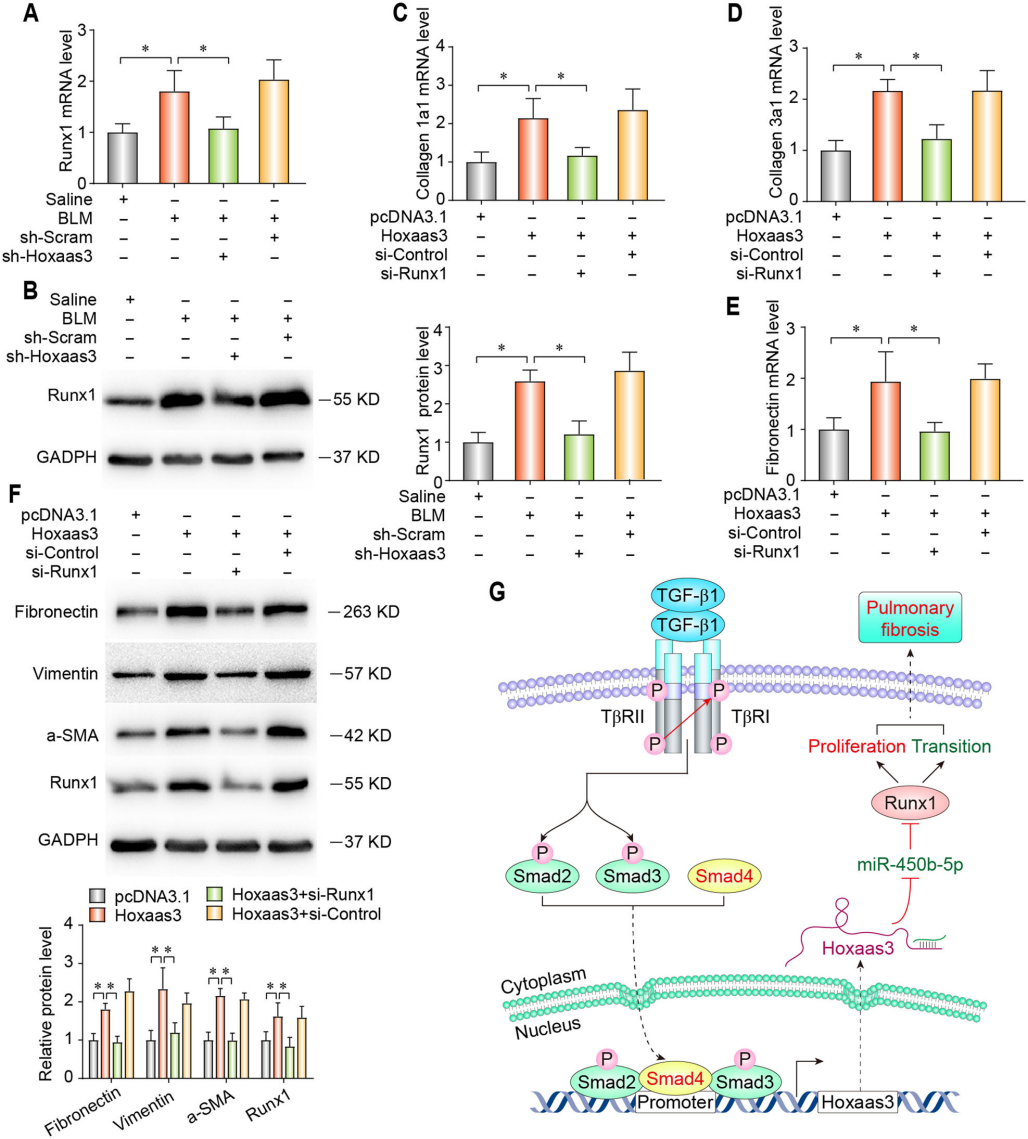
Hoxaas3 contributes to PF by regulating the Runx1. **a**, **b** qPCR and western blot analysis of Runx1 mRNA (**a**) and protein (**b**) level in the lungs of BLM-treated mice with Hoxaas3 inhibition (*n* = 6). (**c**–**e**) qPCR analysis of mRNA levels of collagen 1a1 (**c**), collagen 3a1 (**d**) and Fibronectin (**e**) (*n* = 3). **f** Western blot analysis of expression of Fibronectin, Vimentin, a-SMA, and Runx1 (*n* = 3). **g** Schematic diagram of the molecular mechanisms underlying the TGF-β1/Smad4-Hoxaas3–miR-450b-5p–Runx1 axis-mediated regulation of lung fibrosis (*n* = 3). (All data are presented as mean ± SD, *P < 0.05).

Hoxaas3 inhibition reversed the upregulated mRNA expression of Runx1 in BLM-treated mice (Fig. 8a). Western blot analysis suggested that Hoxaas3 inhibition alleviated the protein level of Runx1 in BLM-induced mice (Fig. 8b). Then, we found Runx1 inhibition attenuated the increase of the mRNA level for collagen-1a1 (Fig. 8c), collagen-3a1 (Fig. 8d), and fibronectin (Fig. 8e) in Hoxaas3-induced fibrogenesis, along with the expression of fibronectin, vimentin, and a-SMA by western blot (Fig. 8f). Meanwhile, it is similar to the effect of miR-450b-5p upregulation.

## Discussion

Several lncRNAs have been discovered in PF through transcriptome sequencing and microarray analysis, etc. The mechanisms based on lncRNAs are one of the hot topics for PF at present. For example, lncPFAL was discovered to promote PF by competitively binding miR-18a to regulate CTGF^17^. Song et al. investigated a novel lncITPF, which was upregulated in a TGF-β1-smad2/3-dependent manner, promotes PF through regulating hnRNP-L and ITGBL1, and interestingly, lncITPF was also correlation with the clinicopathological characteristics of IPF patients^18^. Zhao et al. found lncPFAR contributed to PF through regulating the YAP1-Twist axis and playing the role of molecular sponge for miR-138^19^. Jiang et al. showed lncPFRL contributed to the progression of PF by regulating miR-26a and Smad2^20^. Liu et al. discovered a novel lnc-PCF, promoted PF by targeting miR-344a-5p and map3k11^21^.

Hoxaas3 belongs to Hox gene cluster, regulating embryonic development, hematopoietic lineage, and differentiation, which is a group of highly homologous transcription factors^22,23^. Zhang et al. detected Hoxaas3 increased in the pulmonary blood vessels and the pulmonary artery smooth muscle cells of mice under hypoxic status; meanwhile, Hoxaas3 level raised via histone H3 Lysine 9 acetylation, regulating cell cycle distribution^24^. In this study, we firstly found Hoxaas3 was up-regulated in PF, and promoted fibrogenesis, indicating that Hoxaas3 might be a useful biomarker and a candidate drug target for IPF.

TGF-β1 is involved in a variety of cellular processes, including proliferation, apoptosis, death, differentiation, angiogenesis, and motility^25–27^. TGF-β1 activated its downstream molecules, phosphorylation of Smad2/3, to exert biological functions. In addition, phosphorylation of Smad2/3 binding with Smad4 to form a Smads complex, then get into the nucleus to combine target genes and control its expression. It is known that TGF-β1 plays an important role in PF regulation^28–30^. However, the specific function of Smad4 in PF remains largely unclear. Considering the important role of Smad4 in TGF-β1/Smads signaling pathway, in this study, Smad4 was found to bind to the promoter sequence of Hoxaas3 to enhance its expression.

Cumulating evidence demonstrates that miRNAs play prominent roles in IPF. Several dysregulated miRNAs have been found in the human lungs as well as in animal models with PF^31,32^. These abnormal miRNAs activated and differentiated fibroblasts to myofibroblasts, leading to proliferation and migration of fibroblasts, and excessive ECM accumulation^33^. For example, miR-154^32^ and miR-21^34^ have been reported to be upregulated in PF and led to PF. In contrast, miR-101^31^, miR-9-5p^35^, and miR-18a-5p^36^ were downregulated and played an anti-fibrotic role in PF. In the previous report, miR-450b-5p level was reduced with TGF-β1, a new mechanism to inhibit myogenic differentiation of rhabdomyosarcoma^37^. LncRNA and miRNA often regulate each other in a ceRNA model. In the present study, miR-450b-5p is downregulated in TGF-β1 treatment of lung fibroblasts and pulmonary tissues of BLM-treated mice. The expression and activity of miR-450b-5p were regulated by Hoxaas3, acting as a ceRNA in PF. Knockdown of miR-450b-5p promoted lung fibroblast, whereas upregulation of miR-450b-5p reduced TGF-β1-induced fibrogenesis in CMLF. miR-450b-5p played an anti-fibrotic role in PF.

Runt-related transcription factor 1 (Runx1), one of RUNX family members, including Runx1, Runx2, and Runx3, a family of evolutionarily conserved transcription factors, play a key function in a variety of diseases, such as tumors^38^, pulmonary arterial^39^ hypertension, cardiac remodeling^40^, etc. Runx1 has been shown to regulate EMT via promoting the Wnt/β-catenin signaling pathway in colorectal cancer^41^. In addition, Runx1 played important role in liver fibrosis of non-alcoholic steatohepatitis patients^42^. Interestingly, Runx1 was found to regulate EMT marker genes in renal tubular epithelial cells, and deletion of Runx1 could attenuate renal fibrosis^43^. This study indicated miR-450b-5p reduced the Runx1 level at the transcriptional levels. In addition, Runx1 inhibition eliminates the profibrotic function of miR-450b-5p silencing. These results indicated that Runx1 was one of the targets of miR-450b-5p and mediated its anti-fibrotic effect.

In conclusion, our study showed the role and mechanism of the TGF-β1/Smad4-Hoxaas3–miR-450b-5p–Runx1 axis for a better understanding of PF (Fig. 8g), and demonstrated Hoxaas3 maybe a new diagnostic biomarker or potential therapeutic target for IPF.

## Materials and methods

### Primary tissue samples

Lung tissues from 18 IPF patients and 18 normal lung histology samples were obtained from Tissue Specimen Bank of the First Affiliated Hospital, College of Medicine, Zhejiang University. All participants provided written informed consent. The Ethics Committee of the First Affiliated Hospital, College of Medicine, Zhejiang University, approved this patient study.

### Animal model and cell culture

The animal studies were approved by the Institutional Animal Care and Use Committee of Zhejiang University, Hangzhou, China. Male 6–8-week-old C57BL/6 mice were used in this study. Anesthetized mice received 5 mg/kg bleomycin (BLM, Nippon Kayaku, Tokyo, Japan) or an equal volume of sterile saline via intratracheal administration. The number of mice in each group was six.

In a separate set of experiments, mice were treated with either Lentivirus particles expressing nonsense short hairpin RNA (sh-Scramble) or shRNA targeting Hoxaas3 (sh-Hoxaas3) through intratracheal injection at a dose of 1 × 10 ^11^ vector genomes/mice, 3 day after BLM administration. On days 7, 14, 21, and 28, mice were sacrificed; lung tissue specimen were gathered and then frozen in liquid nitrogen for further studies.

Cultured mouse lung fibroblasts (CMLF) were isolated from 2-day-old C57BL/6 mice, as described previously^17^. Lung tissues were cut into small pieces, digested with 0.25% trypsin following constituted shaking for about 90 min, and then passed through a 200-mesh cell strainer sieve. The cell was centrifuged and resuspended in DMEM supplemented with 10% FBS, 100 U/mL penicillin, and 100 mg/mL streptomycin. CMLF was seeded into culture plates. Further experiments were performed when the cells were in good condition.

### Hematoxylin and Eosin (H&E) Staining and Masson’s Trichrome Staining

Lung tissues were fixed in 4% paraformaldehyde for 24 h, and then embedding in paraffin, 4-mm sections were prepared and stained with H&E and Masson’s trichrome for histopathologic examination. The fibrotic area was quantified with Image Pro Plus 6.0.

### Measurement of Hydroxyproline content

Matrix proteins in lung tissue were determined by Hydroxyproline (HYP) detection kit (Nanjing Jiancheng Institute of Bioengineering, China), following the manufacturer’s instructions. Briefly, the lung tissues were prepared, and then the mixture of oxidizing reagents was added and cultured at room temperature for 20 min. Next, the developer was added and incubated at 37 C for 5 min. Finally, the absorbance was measured at 560 nm. Data are expressed as micrograms of HYP per milligram dry weight of lung tissue.

### Western blot and quantitative real-time reverse-transcription PCR

Western blot and quantitative real-time reverse-transcription PCR (qPCR) were performed as described previously^15,44^. Lysates were generated, and total protein was separated by standard SDS PAGE, followed by transfer to polyvinylidene fluoride (PVDF) membranes. The membranes were then washed and blocked before incubation with the primary antibodies (listed in Table S1). Reactions were detected using enhanced chemiluminescence assays. GADPH was used as a control.

All procedures were performed according to the manufacturer’s instructions. Total RNA was extracted using an Ultrapure RNA kit (CWbio, Co., Ltd., Beijing, China). RNA was reverse-transcribed into cDNA using iScript cDNA Synthesis kits (Bio-Rad Laboratories, Hercules, CA, USA). Quantitative real-time PCR (qPCR) was performed using the SYBR-Green PCR kit (Applied Biosystems, Foster City, CA, USA). GADPH or U6 were used as a loading control.

### Ethynyl deoxyuridine assay

Ethynyl Deoxyuridine (EDU) was performed as described previously^15^. Click-iT EDU Imaging Kit (Invitrogen, Carlsbad, CA, USA) was used according to the manufacturer’s protocol. Briefly, cells were incubated with the IC50 of cisplatin for 48 h and then with 10 μM EDU for 2 h before fixation, permeabilization, and staining for EDU. Nuclei were counterstained with DAPI for 5 min.

Wound healing assay was performed as described previously^17^.

### Chromatin immunoprecipitation assays

Chromatin immunoprecipitation (ChIP) assays were performed using a ChIP Assay Kit according to the manufacturer’s instructions (Cell Signaling Technology, SimpleChIP Plus Enzymatic Chromatin IP Kit (Magnetic Beads) #9005). The genomic DNA fragments were immunoprecipitated with antibodies against Smad4 and normal rabbit IgG (listed in Table S1) at 4 °C for 3 h. After crosslinking reversal and DNA cleanup, the purified and precipitated DNA was analyzed by qPCR. The primers targeting the Hoxaas3 promoter region were 5′-CAATTGATCCCGGATTCCACA-3′ and 5′- CACCCTTTCTGAAGGCTAAGTG -3′.

### Plasmid construction and transfection

For Hoxaas3 overexpression, the full-length Hoxaas3 cDNA was cloned into vector pcDNA3.1, and stable clones were obtained with G418. Empty vector pcDNA3.1 was used as a negative control. For Hoxaas3 knockdown, shRNAs targeting Hoxaas3 (sh-Hoxaas3) and a scrambled shRNA (sh-Scram) used as negative control were synthesized. The miR-450b-5p mimics/miR-Ctrl, miR-450b-5p inhibitors/NC, si-Smad4/si-Control, si-Runx1/si-Control and respective negative controls were purchased from GenePharma (Shanghai, China). Transfection using Lipofectamine 3000 (L3000015, Thermo Fisher Scientific, Waltham, MA, USA) was performed according to a standard protocol.

### Luciferase reporter assay

For the luciferase reporter assay, oligonucleotides containing the target sequence of Hoxaas3/Runx1 and its mutant form devoid of the miR-450b-5p-binding site were amplified and cloned into a pGL3 vector immediately downstream of the luciferase gene. To identify the influence of Hoxaas3/ Runx1 on miR-450b-5p activity, an miR-450b-5p sensor reporter was constructed. Lung fibroblasts or HEK293 cells were transfected with the luciferase constructs and miRNAs using Lipofectamine 3000. Luciferase activity was measured at 36 h post transfection with a Dual-Luciferase assay system (Promega, Madison, WI, USA) according to the manufacturer’s protocols.

### Statistical analysis

Statistical analyses were performed using GraphPad Prism software version 5.0 (GraphPad Software, Inc., La Jolla, CA, USA) and SPSS version 19.0 (SPSS Inc., Chicago, IL, USA)). Unpaired Student’s *t* test was used for experiments comparing two groups, whereas one-way ANOVA with Student-Newman-Keuls post hoc test was applied for experiments comparing three or more groups. The mean Standard Deviation (SD) of at least three independent experiments was determined. *P*-values < 0.05 were considered statistically significant.

## Supplementary information

Figure Legend - Supplement

Table S1- Supplement

Figure S1

Figure S2

Figure S3

Figure S4
