# Supplementary material for: LncRNA Hoxaas3 promotes lung fibroblast activation and fibrosis by targeting miR-450b-5p to regulate Runx1
Source: Cell Death Dis. 2020 Aug 26;11(8):706. doi: 10.1038/s41419-020-02889-w (PMC7450059; doi:10.1038/s41419-020-02889-w)
Supplement: Supplementary file 1 — Figure Legend - Supplement [file 41419_2020_2889_MOESM1_ESM.docx]

**Fig. S1 Establishment of BLM-induced pulmonary fibrosis in mice. (a)** Representative images of H&E and Masson staining of C57BL/6 mice lung tissues, Scale bars, 200 μm. **(b)** Fibrotic area was statistically measured using Image-Pro Plus. **(c, d, e)** qPCR analysis of mRNA levels of collagen 1a1 **(c)**, collagen 3a1 **(d)** and Fibronectin **(e)** in mice. **(f)** Western blot analysis of expression of Fibronectin and a-SMA in mice. All data are presented as mean ± SD, n=6, *P < 0.05.

**Fig. S2 Representative images** **of H&E and Masson staining in BLM-induced mice. (a)** Representative images of H&E and Masson staining of heart from BLM-induced mice. **(b)** Representative images of H&E and Masson staining of liver from BLM-induced mice. **(c)** Representative images of H&E and Masson staining of kidney from BLM-induced mice. All data, n=6.

**Fig. S3 Silencing Hoxaas3 alleviates TGF-β1-induced lung fibroblast activation. (a, b, c)** qPCR analysis of mRNA levels of collagen 1a1 **(a)**, collagen 3a1 **(b)**, and Fibronectin **(c)** after treatment with TGF-β1 together with Hoxaas3 inhibition in cultured lung fibroblasts. **(d)** Western blot analysis of expression of Fibronectin, Vimentin and a-SMA in TGF-β1-treated lung fibroblasts after Hoxaas3 silencing. **(e)** Proliferative capacity was analyzed using an EDU assay. **(f)** Wound-healing assays were performed to examine cell migration. All data are presented as mean ± SD, n=3, *P < 0.05.

**Fig. S4 Overexpression of miR-450b-5p alleviates TGF-β-induced fibrogenesis. (a, b, c)** qPCR analysis of mRNA levels of collagen 1a1 **(a)**, collagen 3a1 **(b)**, and Fibronectin **(c)** after treatment with TGF-β1 together with miR-450b-5p mimic in cultured lung fibroblasts. **(d)** Western blot analysis of expression of Fibronectin, Vimentin and a-SMA in TGF-β1-treated lung fibroblasts after transfection with miR-450b-5p mimic. **(e)** Proliferative capacity was analyzed using an EDU assay. **(f)** Wound-healing assays were performed to examine cell migration. All data are presented as mean ± SD, n=3, *P < 0.05.
