# Supplementary material for: LncRNA Hoxaas3 promotes lung fibroblast activation and fibrosis by targeting miR-450b-5p to regulate Runx1
Source: Cell Death Dis. 2020 Aug 26;11(8):706. doi: 10.1038/s41419-020-02889-w (PMC7450059; doi:10.1038/s41419-020-02889-w)
Supplement: Supplementary file 2 — Table S1- Supplement [file 41419_2020_2889_MOESM2_ESM.docx]

**Table S1. Antibodies used for western blotting (WB) and ChIP.**

| **Antibody** | **Company** | **Application** |
| --- | --- | --- |
| Fibronectin | Proteintech (# 15613-1-AP) | WB |
| a-SMA | Cell Signaling Technology (# 9245) | WB |
| Vimentin | Cell Signaling Technology (# 5741) | WB |
| Runx1 | Cell Signaling Technology (# 8529) | WB |
| Smad4 | Cell Signaling Technology (# 46535) | WB and ChIP |
| IgG | Cell Signaling Technology (#3900) | ChIP |
| GADPH | Cell Signaling Technology (# 5174) | WB |
