# Supplementary figures and images for: LncRNA Hoxaas3 promotes lung fibroblast activation and fibrosis by targeting miR-450b-5p to regulate Runx1
Source: Cell Death Dis. 2020 Aug 26;11(8):706. doi: 10.1038/s41419-020-02889-w (PMC7450059; doi:10.1038/s41419-020-02889-w)

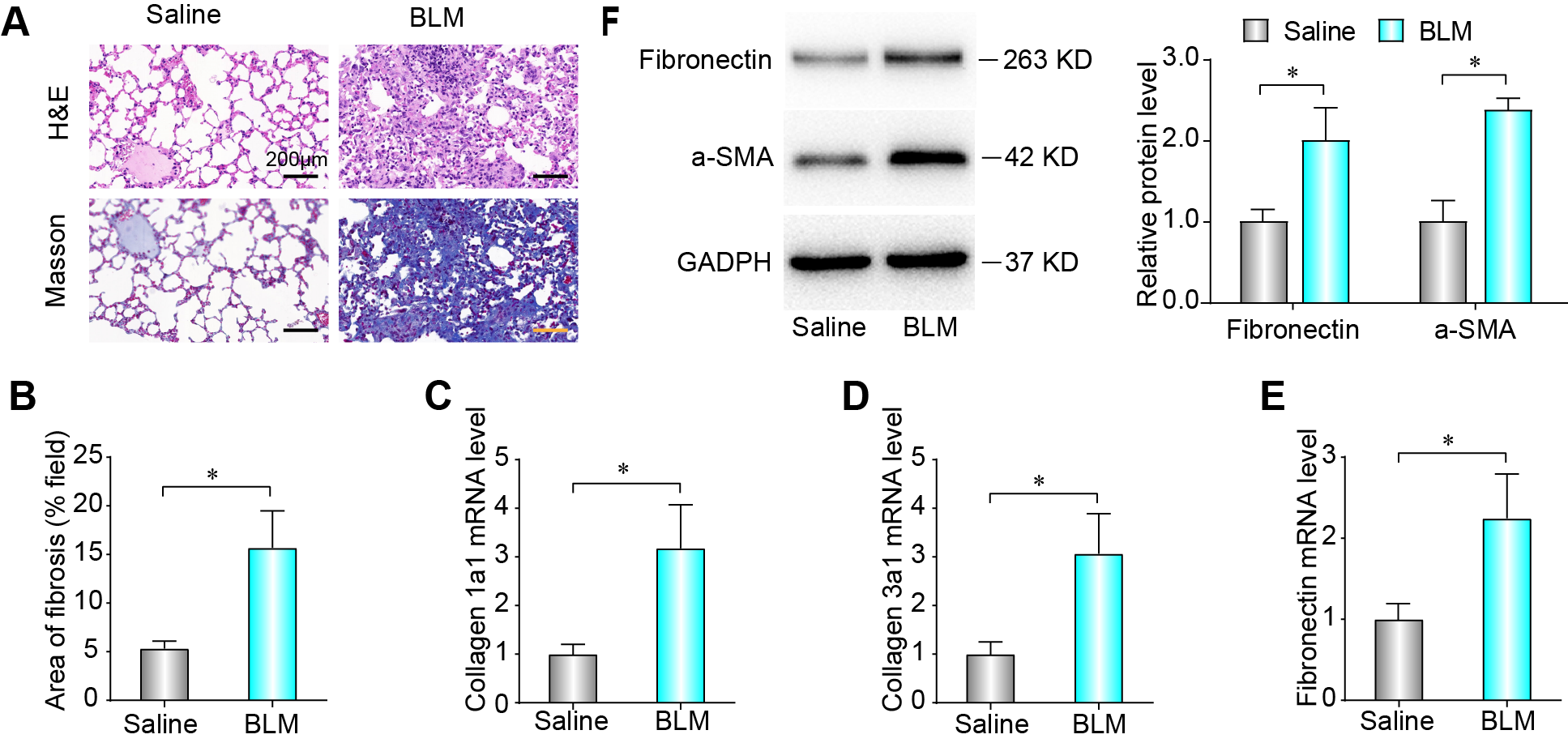

Supplement: Supplementary file 3 — Figure S1 [file 41419_2020_2889_MOESM3_ESM.png]

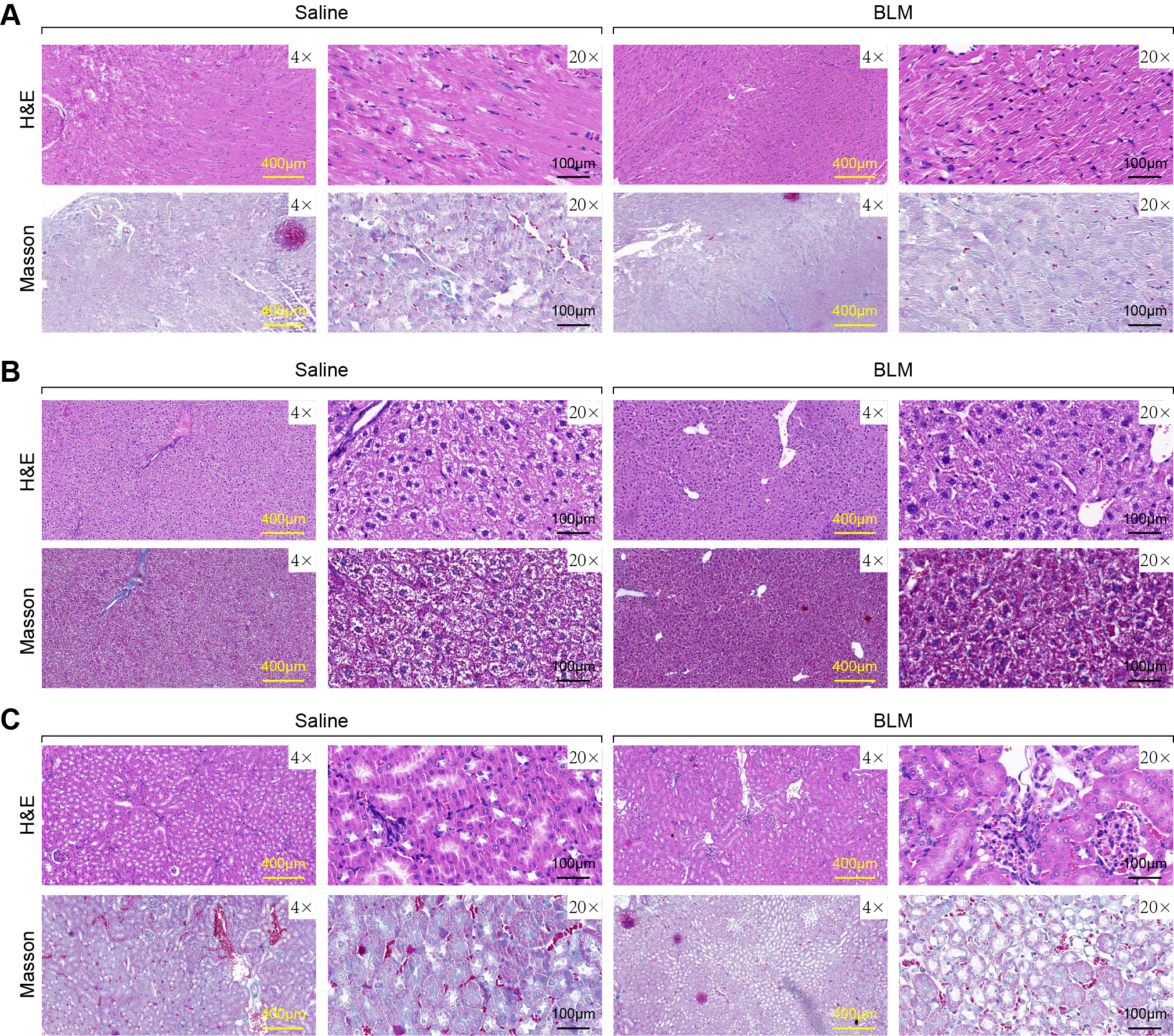

Supplement: Supplementary file 4 — Figure S2 [file 41419_2020_2889_MOESM4_ESM.png]

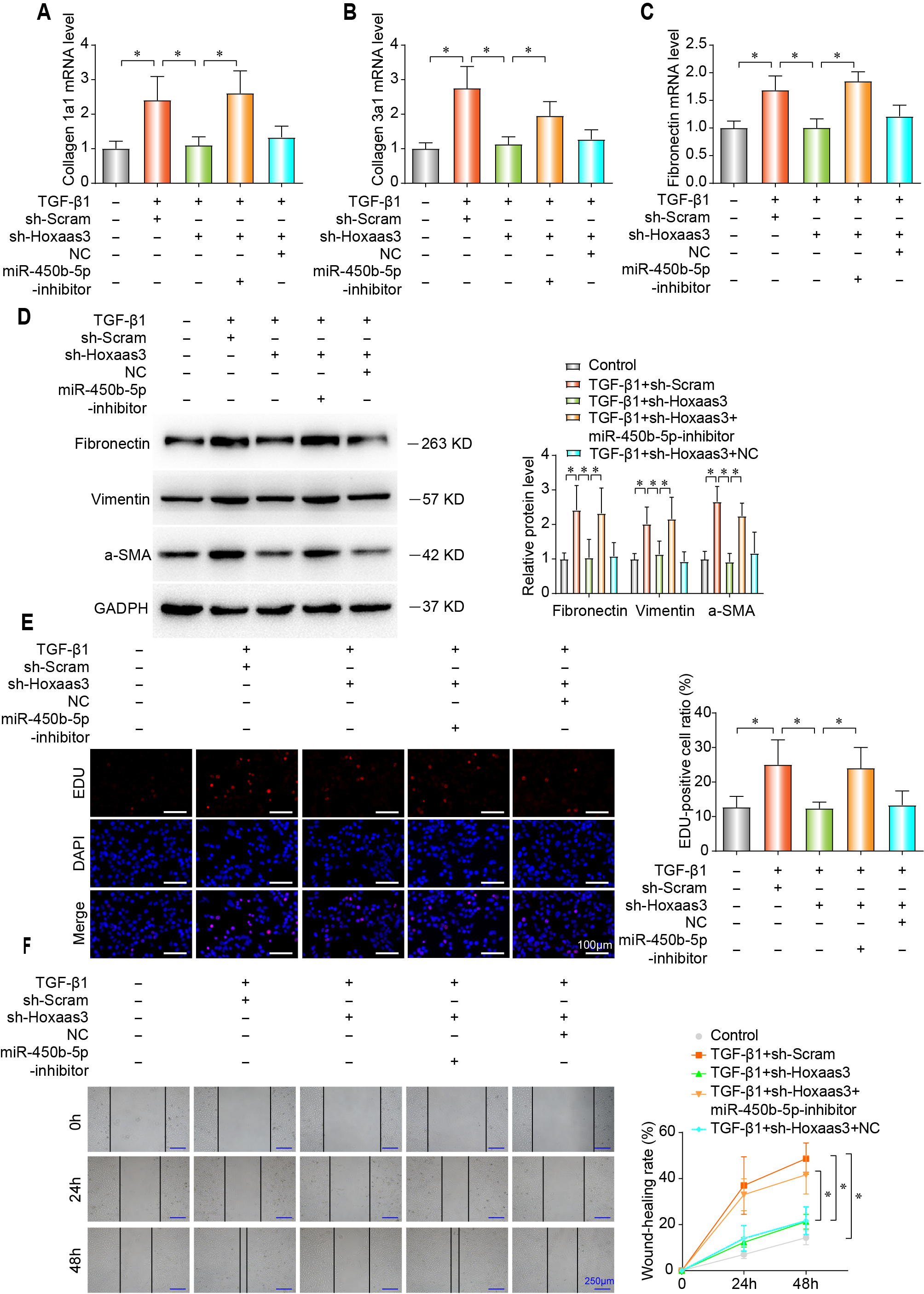

Supplement: Supplementary file 5 — Figure S3 [file 41419_2020_2889_MOESM5_ESM.png]

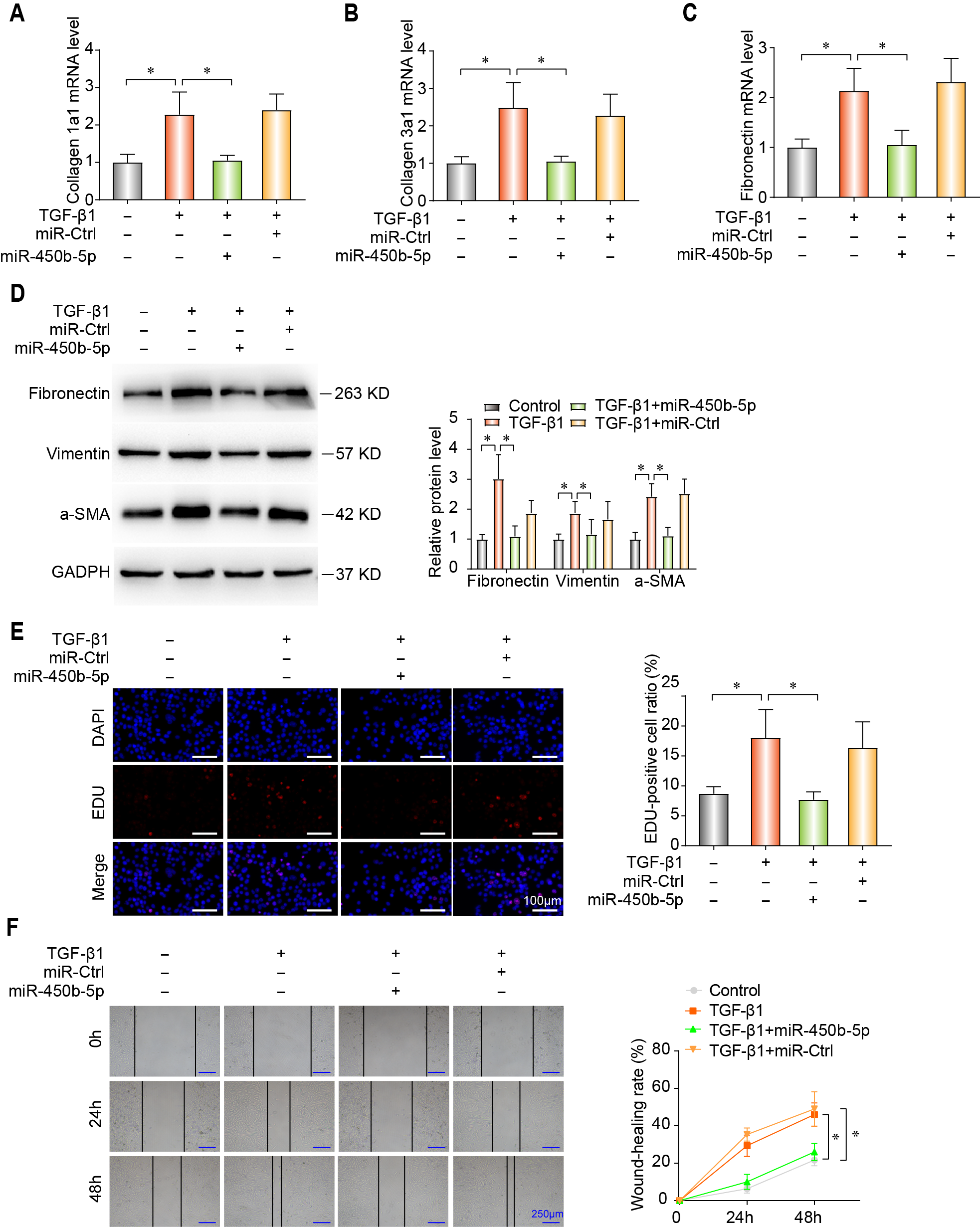

Supplement: Supplementary file 6 — Figure S4 [file 41419_2020_2889_MOESM6_ESM.png]
